# Aberrant DNA Methylation of Matrix Remodeling and Cell Adhesion Related Genes in Pterygium

**DOI:** 10.1371/journal.pone.0014687

**Published:** 2011-02-16

**Authors:** Andri K. Riau, Tina T. Wong, Sharon N. Finger, Shyam S. Chaurasia, Ai Hua Hou, Silin Chen, Shang Juan Yu, Louis Tong

**Affiliations:** 1 Ocular Wound Healing and Therapeutics Laboratory, Singapore Eye Research Institute, Singapore, Singapore; 2 Singapore National Eye Center, Singapore, Singapore; 3 Department of Ophthalmology, Yong Loo Lin School of Medicine, National University of Singapore, Singapore, Singapore; 4 School of Materials Science and Engineering, Nanyang Technological University, Singapore, Singapore; 5 Duke-NUS Graduate Medical School, Singapore, Singapore; Duke-National University of Singapore Graduate Medical School, Singapore

## Abstract

**Background:**

Pterygium is a common ocular surface disease characterized by abnormal epithelial and fibrovascular proliferation, invasion, and matrix remodeling. This lesion, which migrates from the periphery to the center of the cornea, impairs vision and causes considerable irritation. The mechanism of pterygium formation remains ambiguous, and current treatment is solely surgical excision, with a significant risk of recurrence after surgery. Here, we investigate the role of methylation in DNA sequences that regulate matrix remodeling and cell adhesion in pterygium formation.

**Methodology/Principal Findings:**

Pterygium and uninvolved conjunctiva samples were obtained from the same eye of patients undergoing surgery. The EpiTYPER Sequenom technology, based on differential base cleavage and bisulfite sequencing was used to evaluate the extent of methylation of 29 matrix and adhesion related genes. In pterygium, three CpG sites at −268, −32 and −29 bp upstream of transglutaminase 2 (TGM-2) transcription initiation were significantly hypermethylated (*p*<0.05), whereas hypomethylation was detected at CpGs +484 and +602 bp downstream of matrix metalloproteinase 2 (MMP-2) transcription start site, and −809, −762, −631 and −629 bp upstream of the CD24 transcription start site. RT-qPCR, western blot and immunofluorescent staining showed that transcript and protein expression were reduced for TGM-2 and increased for MMP-2 and CD24. Inhibition of methylation in cultured conjunctival epithelial cells increased these transcripts.

**Conclusions/Significance:**

We found regions of aberrant DNA methylation which were consistent with alteration of TGM-2, MMP-2, and CD24 transcript and protein expression, and that inhibition of methylation in cultured cells can increase the expression of these genes. Since these genes were related to cell adhesion and matrix remodeling, dysregulation may lead to fibroblastic and neovascular changes and pterygium formation. These results have implications for the prognostication of pterygium in clinical practice, for example, detection of epigenetic changes may have a role in predicting post surgical recurrence of aggressive lesions.

## Introduction

Pterygium is a common fibrovascular ocular surface disease characterized by migration of wedge-shaped abnormal tissue from bulbar conjunctiva onto the cornea. Morbidity incurred by pterygium includes irritation, redness, and visual impairment in instances, such as visual occlusion induced by a large pterygium that has migrated to the visual axis over the central cornea, and irregular astigmatism or impaired tear film regularity induced by pterygium. The pathogenesis of this disease, however, remains ambiguous. Compelling evidence that UV-mediated limbal damage triggers this pathogenesis has been reviewed [1]. Other causes proposed include aberrant wound healing mechanisms [2]–[4], genetic instability [5], stem cell dysfunction [6], metabolic disorder [7], and neuronal dysfunction [8].

Epigenetic modifications of gene expression are known to play an established role in the development of human cancers [9]. Both DNA hypo- and hypermethylation within promoter-rich cytosine guanine dinucleotide (CpG) islands have given rise to cancers [10], [11]. Additionally, DNA methylation has long been suspected to mediate some of the effects of aging, environmental exposures and lifestyle factors on risk of non-neoplastic diseases [12], [13].

Epigenetic changes have been implicated in the control of wound healing [14], [15]. For example, wound healing and fibrosis in the liver is mediated by critical processes controlled by DNA methylation [14]. DNA methylation in mammals occurs predominantly at the CpG islands, and approximately 60–90% of the dinucleotides are modified [16]. This DNA modification in gene promoters causes transcriptional repression by directly interfering with the binding of transcription factors to DNA, or alternatively, gene repression occurs because methylated DNA attracts inhibitory proteins that block access to the factors responsible for induction of the gene [16]. Within the field of epigenetics, DNA methylation patterns are fast becoming an increasing interest to the scientific community. Changes in DNA methylation are a dynamic process and the resulting patterns are tightly associated to disease. In the case of the ocular surface, such changes are more likely to be spatially confined to one part of the tissue to cause disease, compared to changes in genomic sequences, which are ubiquitous and tend to be present in both the diseased and normal tissue.

Promoter methylation has been known to play important roles in genes related to wound healing. For example, transglutaminase 2 (TGM-2) is involved in corneal wound healing [17], and is regulated epigenetically by promoter methylation [18]. TGM-2, a family member of transglutaminases, is a ubiquitously expressed protein comprised of a single ∼76 kDa polypeptide. It exhibits calcium-dependent protein cross-linking activity [19], and enhances cell adhesion and intracellular signaling, and extracellular matrix (ECM) remodeling [20].

Wound healing processes are also mediated by the matrix metalloproteinases (MMP) such as MMP-2, which is primarily secreted by keratocytes in the cornea and mediates long-term stromal remodeling and basement membrane synthesis [21]. This protein has been shown to be increased during corneal repair [21], [22], and is known to be regulated by DNA methylation [23].

Recently, a cell adhesion molecule that has never been previously associated with pterygium, CD24, was reported to be upregulated and localized in the nuclei in pterygium epithelium which suggest that cell adhesion properties may be disrupted [24]. CD24, also known as heat-stable antigen in the mouse, is a glycoprotein of heterogeneous molecular weight ranging from 30 to 70 kDa [25]. CD24 has been repeatedly detected in gene expression profiling to identify genes which expression correlates with tumorigenesis and tumor progression [26]–[28]. Moreover, CD24 has been demonstrated to promote tumor cell invasiveness in animal model [29] and in vitro [30].

Despite the important findings on promoter methylation and wound healing, there has been no reported study on the aberrant methylation in pterygium. We have previously investigated the genes specifically involved in wound healing mechanisms such as TGM-2, MMP-2, and CD24 in pterygium [24]. Here, we report for the first time the role of methylation in the pathogenesis of pterygium, by profiling the methylation status of TGM-2, MMP-2, and CD24 gene promoters and the effect of methylation in cultured ocular surface cells.

## Results

### DNA methylation analysis

**Figure 1:**
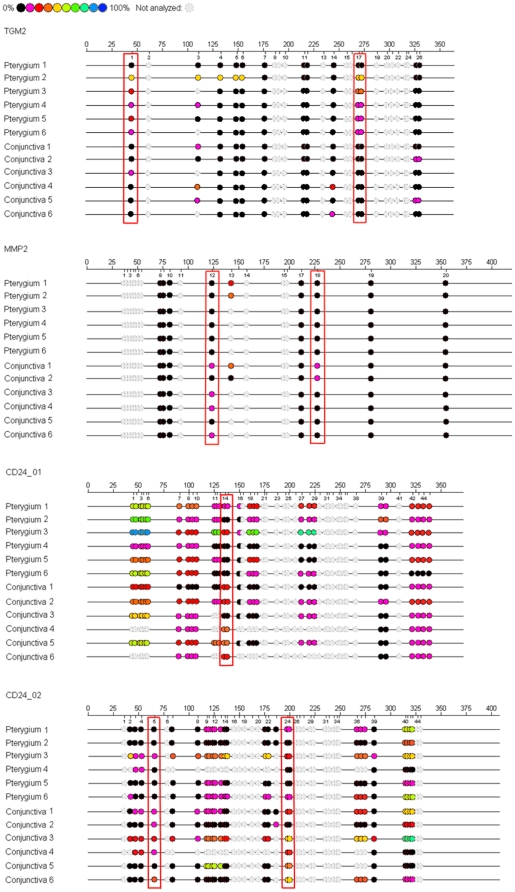
Epigrams showing the nucleotide sequences used in the EpiTYPER Sequenom study. It was obtained from genes known to be important for matrix remodeling and cell adhesion. The brackets in red indicated the differentially methylated CpG sites that were consistent with transcript level changes in pterygium.

The methylation status at the promoters of TGM-2, MMP-2, and CD24 genes was significantly different between pterygium and uninvolved conjunctiva samples in at least one CpG unit. Since DNA methylation is generally a form of negative regulation of gene transcription [31], a relatively higher level of methylation at a particular CpG island was considered to be biologically important if this corresponded to a relatively lower expression of the transcript in this tissue, and vice versa. Using this approach, out of 29 tested genes, the methylation status of the CpG units of the following 3 genes was considered to be biologically important: CpG 1 and 17/18 at the TGM-2 promoter; CpG 14/15 at the CD24 promoter (CD24_01); CpG 5 and 24/25 at the CD24 promoter (CD24_02); and CpG 12 and 18 at the MMP-2 promoter. The numbers of the CpG refers to the numbered CpG units along the EpiTYPER target sequences. The relative methylation level of pterygium and conjunctival sample in each patient, as well as the mean methylation are tabulated in **Table 1**. **Figure 1** shows the epigrams highlighting the CpGs that were methylated to a significantly different extent between pterygium and uninvolved conjunctiva.

**Table 1:** Methylation levels of TGM-2, MMP-2, and CD24 in pterygium and conjunctiva.

	CpG	Patient	Methylation level		Mean methylation±SD			Transcription
Sample	position*	no.	P	C	P	C	p-value	level***
TGM-2	CpG 1	1	0.11	0.12	0.260±0.131	0.100±0.085	0.015	TGM-2 was
		2	0.49	0.05				downregulated
		3	0.31	0.25				0.479 fold
		4	0.20	0.07				in pterygium
		5	0.26	0				relative to
		6	0.19	0.11				conjunctiva
	CpG 17/18	1	0.13	0.04	0.256±0.144	0.060±0.041	0.005	
		2	0.46	0.06				
		3	0.42	0.13				
		4	0.16	0.01				
		5	0.20	0.08				
		6	0.17	0.04				
MMP-2	CpG 12	1	0.01	0.17	0.042±0.038	0.162±0.022	2.85×10^−5^	MMP-2 was
		2	0.01	0.13				upregulated
		3	0.09	0.17				1.324 fold
		4	0.09	0.17				in pterygium
		5	0.02	0.14				relative to
		6	0.03	0.19				conjunctiva
	CpG 18	1	0.01	0.07	0.017±0.018	0.057±0.030	0.009	
		2	0.04	0.10				
		3	0	0.01				
		4	0.04	0.06				
		5	0	0.04				
		6	0.01	0.06				
CD24_01**	CpG 14/15	1	0.17	0.29	0.120±0.123	0.337±0.122	0.006	CD24 was
		2	0.10	0.12				upregulated
		3	0.33	0.43				3.42 fold in
		4	0	0.45				pterygium
		5	0	0.40				relative to
		6	0.12	0.33				conjunctiva
CD24_02**	CpG 5	1	0.14	0.15	0.078±0.077	0.243±0.097	0.004	
		2	0.02	0.23				
		3	0.18	0.35				
		4	0	0.22				
		5	0.01	0.14				
		6	0.12	0.37				
	CpG 24/25	1	0.20	0.28	0.142±0.142	0.372±0.154	0.011	
		2	0.04	0.13				
		3	0.35	0.51				
		4	0.01	0.33				
		5	0.01	0.45				
		6	0.24	0.53				

P refers to pterygium and C refers to conjunctiva.

*The position of the CpG refers to the numbered CpG units along the EpiTYPER target sequences.

**More than one sequences were used for this promoter and found to be differentially methylated.

***Transcription levels were based on our previously published work (see reference #24).

Supplementary **Figure S1** shows three specific CpG islands that were significantly hypermethylated in pterygium in comparison to conjunctiva tissue, located at the −268, −32 and −29 positions of TGM-2 transcription start site (NCBI accession no. U13920). These positions corresponded to −367, −131 and −128 bp, respectively, upstream of the translation start site. **Figure S2** illustrates a differentially hypomethylated CpG unit in pterygium tissue, which was located at the +484 and +602 bp downstream of MMP-2 transcription start site (NCBI accession no. NM_004530.4). In **Figure S3A**, a CpG unit located −698 bp upstream of CD24 transcription start site (NCBI accession no. Y14692), corresponding to −809 bp upstream of the start codon, was shown to be significantly hypomethylated in the pterygium tissue. Other differentially hypomethylated CpG units were located −762, −631 and −629 bp upstream of the CD24 transcription start site (**Figure S3B**).

### Effect of 5-aza-2′-deoxycytidine treatment

**Figure 2:**
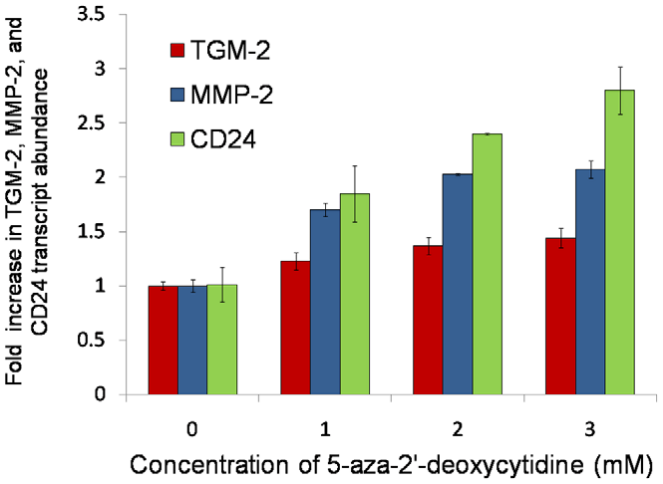
The effect of 5-aza-2′-deoxycytidine (5-aza-dC) on transglutaminase 2 (TGM-2), matrix metalloproteinase 2 (MMP-2) and CD24 transcript levels in human conjunctival epithelial cells. Cultured conjunctival epithelial cells (IOBA-NHC) were treated with 1, 2 or 3 mM of 5-aza-dC.

In order to determine whether DNA methylation might be a general phenomenon that controls cell-specific TGM-2, MMP-2, and CD24 expression in the conjunctiva, we treated spontaneously immortalized human conjunctival epithelial cells (IOBA-NHC) with 5-aza-2′-deoxycytidine (5-aza-dC) and monitored TGM-2, MMP-2, and CD24 mRNA expression. The methyltransferase inhibitor, 5-aza-dC, stimulated the upregulation of TGM-2, MMP-2, and CD24 (**Figure 2**) and demonstrated a dose-dependent effect on the mRNA levels of the three transcripts, with a maximal level of mRNA expressed at an inhibitor concentration of 3 mM.

### TGM-2, MMP-2, and CD24 expression

**Figure 3:**
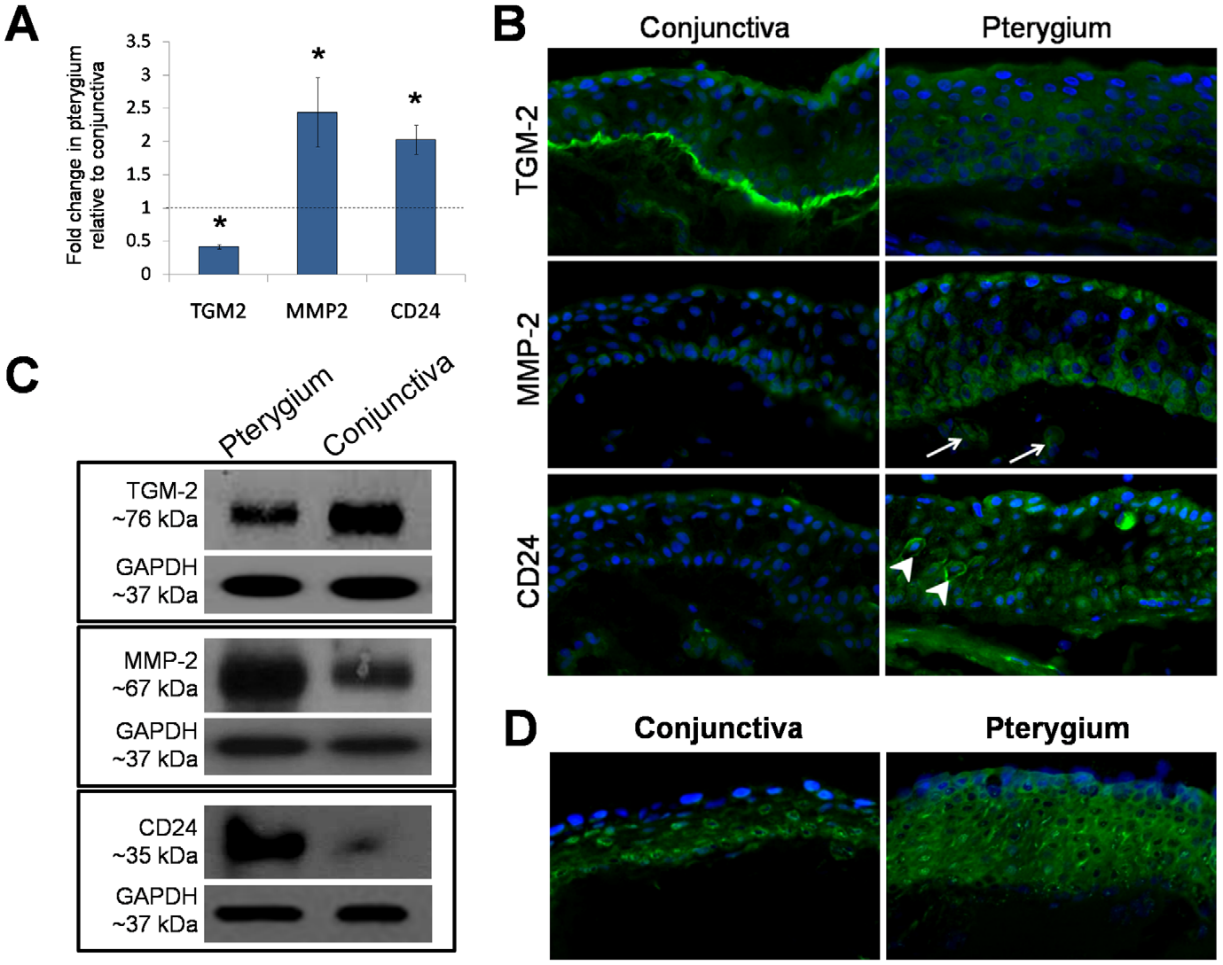
Protein level and localization, as well as transcript level of transglutaminase 2 (TGM-2), matrix metalloproteinase 2 (MMP-2), and CD24 in pterygium compared to conjunctiva. (A) Fold change of TGM-2, MMP-2, and CD24 gene transcript in the pterygium tissue relative to the conjunctiva tissue. Height of the bars represents mean value and error bars represent standard error of the mean. Dashed line represents the transcript levels of the conjunctiva. *p<0.05. (B) Immunofluorescent staining of TGM-2, MMP-2, and CD24. Nuclei were stained with DAPI present in the mounting medium. Arrows indicate fibroblasts that were positive with MMP-2. Arrowheads indicate staining of CD24 in the plasma membrane of epithelial cells. All images were taken at 400X magnification. (C) Western blot analysis of TGM-2, MMP-2, and CD24 proteins expressed in human conjunctiva and pterygium tissue. GAPDH was used as the loading control. (D) In situ zymogram showing gelatinolytic activity in the pterygium and conjunctiva tissue. Images were taken at 400X magnification.

TGM-2, MMP-2, and CD24 gene transcripts were detected in conjunctiva and pterygium tissues. TGM-2 transcript was downregulated by 0.42±0.03 fold (*p*<0.05) in pterygium relative to the conjunctiva. MMP-2 and CD24 transcripts however, were upregulated 2.44±0.52 fold and 2.03±0.22 fold (*p<*0.05), respectively in pterygium compared to normal conjunctiva. A bar graph summarizing the fold differences of these genes in pterygium tissue compared to the conjunctiva tissue is illustrated in **Figure 3A**.

Immunofluorescent staining detected the presence of TGM-2, MMP-2, and CD24 in human conjunctiva and pterygium tissue (**Figure 3B**). No fluorescent signals were detected in negative controls (data not shown). TGM-2 was expressed superficially in the conjunctival epithelium. Strong staining was observed in the basement membrane of the conjunctiva, which was obviously absent in the pterygium.

MMP-2 and CD24 protein expression was attenuated in the normal conjunctiva. MMP-2 was distributed mainly in the basal and a few layers of suprabasal cells in the pterygium epithelium. Some fibroblasts adjacent to the epithelial layer were also stained with the MMP-2 (**Figure 3B**). CD24 was expressed in almost all layers of the pterygium epithelium. Nuclear staining was mainly present in the basal and some suprabasal cells. A few cells were found to be expressed in the plasma membrane (**Figure 3B**).

Western blot further confirmed the expression levels observed in the immunostaining and the mRNA levels detected by the RT-PCR (**Figure 3C**). TGM-2 protein level was relatively lower in the pterygium compared to the conjunctival tissue. In contrast, MMP-2 and CD24 protein levels were both increased in the pterygium compared to the conjunctiva tissue.

In situ zymography showed that pterygium had relatively higher gelatinolytic activity in the epithelium than conjunctiva (**Figure 3D**). No fluorescent signals were detected in sections treated with metalloproteinase inhibitor, 1,10-phenanthroline (data not shown).

## Discussion

In this study, we demonstrate a significant increase in both MMP-2 and CD24 transcript and protein levels in pterygium tissue, with less methylation in the corresponding genomic sequences. In contrast, TGM-2 mRNA and protein levels were reduced in the pterygium tissue.

Previous studies in pterygium have shown MMPs to play a role in the pathogenesis of pterygium [1], [32], [33]. Elevated MMP-2 detected in pterygium has been shown to facilitate the invasive property of pterygium by degrading components of their basement membrane and adjacent stromal matrix [32]. In addition, increased MMP-2 activity in skin fibroblasts has been considered as the key mediator between the increased protease activity and reduced cell adhesion [34].

TGM-2 has been known to cross-link fibronectin, collagen type I, fibrin, and many other ECM proteins after its release from cells exposed to stress or trauma [35]. ECM- bound TGM-2 has been proposed to provide a shield around wounds thereby protecting the structural integrity of the wounded cells from further damage [36]. In addition, a decrease in TGM-2 activity has been reported to result in concomitant attenuation in cell adhesion [37], which may for example, facilitate migration of abnormal pterygium tissue towards the central cornea. Many other known functions of TGM-2 in wound healing that may explain pterygium formation have been reported, including effects on myofibroblasts [38]. Pterygium has been linked to abnormal cell transformation such as epithelial-mesenchymal transition (EMT) to a myofibroblastic phenotype [39]. During EMT, epithelial cells show less intercellular adherence junctions, tight junctions, and desmosomes leading to the loss of cellular polarity. Cytokeratin intermediate filaments are also disassembled to rearrange their F-actin stress fibers to express filopodia and lamellipodia [40]. Transmission electron micrograph showed that basal pterygium epithelial cells had higher cytoplasmic electron density with cytoplasmic fibrils, and the epithelial cells that invaded the underlying stroma no longer showed adhesion complexes and had enlarged intercellular spaces [41]. In addition, co-expression of cytokeratins with α-SMA indicates a classic sign of EMT and the role of myofibroblasts in the progression of pterygium [41].

Numerous studies have linked CD24 with tumorigenesis and tumor progression [26], [28]. CD24 is able to promote tumor cell proliferation and alter the adhesive properties of tumor cells to P-selectin, fibronectin, collagens type I and IV, and laminin [30]. Additionally, cell spreading, motility, and invasiveness are also strongly increased upon CD24 expression [30]. In pterygium, the elevated CD24 expression may cause increased proliferation, motility and invasiveness of pterygium epithelial cells and fibroblasts.

Our study has indicated that the changes in TGM-2, MMP-2, and CD24 expression in the human conjunctival epithelial cells can be regulated by DNA methylation. Concomitantly, previous studies have highlighted the importance of TGM-2 and MMP-2 promoter methylation for the expression of these genes [18], [23], [42], but there has been no previous report concerning the epigenetic regulation of the human CD24.

Demethylation of the TGM-2 promoter at two HpaII tiny fragment (HTF) islands: HTF-1 (+1 to −215 with reference to start of transcription) and HTF-2 (−1315 to −1415), has been linked to tumor cell phenotype [42]. In contrast, we found that the 3 CpG units that were relatively hypermethylated were located at the −268, −32 and −29 bp from the beginning of the transcript, suggesting that hypermethylation upstream of the transcription start site may interfere with binding of some transcription factors, thereby reducing TGM-2 transcription.

Differential methylation of the MMP-2 promoter has been reported in the region up to +733 bp from the transcription start site, in a breast cancer cell line [23]. In this study, we found a hypomethylated CpG unit located +484 and +602 bp downstream of MMP-2 transcription start site in pterygium.

**Figure 4:**
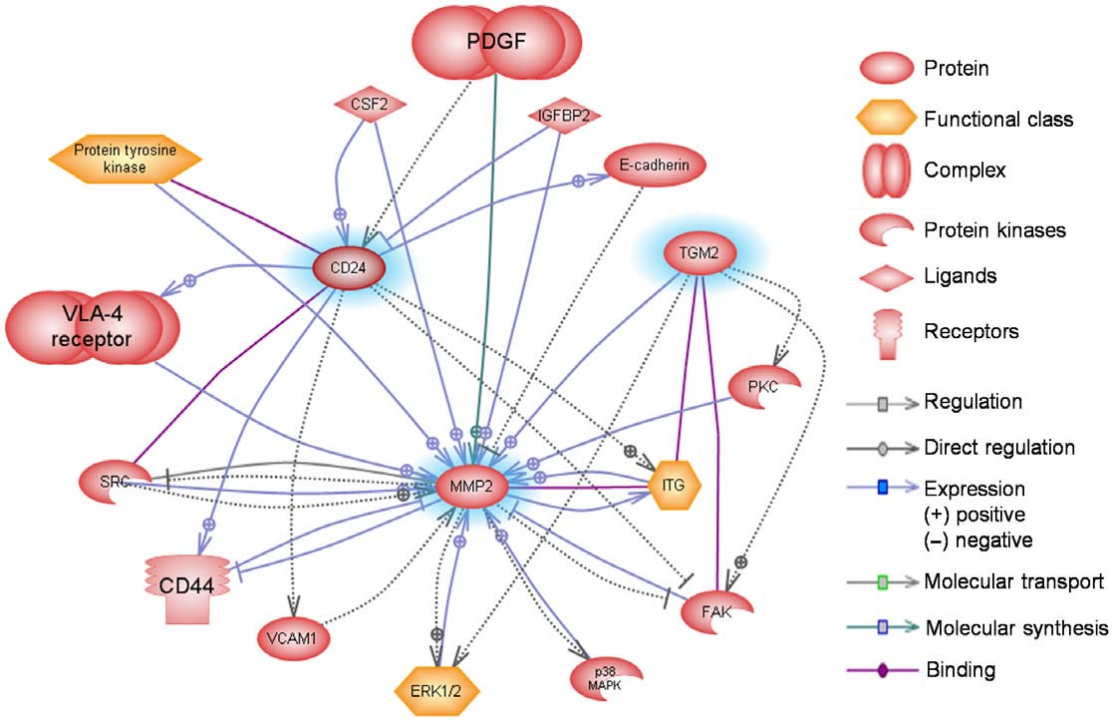
Interacting pathways of transglutaminase 2 (TGM-2), matrix metalloproteinase 2 (MMP-2), and CD24 in pterygium. Selected entities were analyzed with interacting TGM-2, MMP-2, and CD24 pathways by using Ariadne Pathway Studio 6.0.

TGM-2, MMP-2 and CD24 are shown to be linked to one another and other important intracellular signaling molecules either indirectly, or directly, in known biological pathways (**Figure 4**). The molecules in the pathways and the Medline references (PMID numbers) corresponding to the illustrated relationships are tabulated in the supplementary data (**Table S1 and S2**). The observed biological effects of the gene expression changes may be more than expected based on differential methylation of individual genes. For example, TGM-2 can activate MMP-2 in skin fibroblasts [43], increasing the overall biological effect of the pathways mediated by TGM-2 and MMP-2. Another example would be the upregulation of CD24 in microglial cells [44] and MMP-2 in head and neck squamous cell carcinoma [45] by granulocyte-macrophage colony stimulating factor (CSF2). This raises the possibility that critical DNA demethylation may facilitate transcription factor(s) downstream of CSF2 to bind to regulatory sequences of CD24 and MMP-2, with a consequential increase in Wnt/β-catenin signaling, which has been previously reported in pterygium [41]. The canonical Wnt/β-catenin signaling pathway is known as a component to drive EMT in many tumors [46]. In addition, MMP-2 is the downstream gene of the β-catenin signaling pathway [47], and was uniquely expressed in pterygium epithelial cells [1], [32], [33].

**Figure 5:**
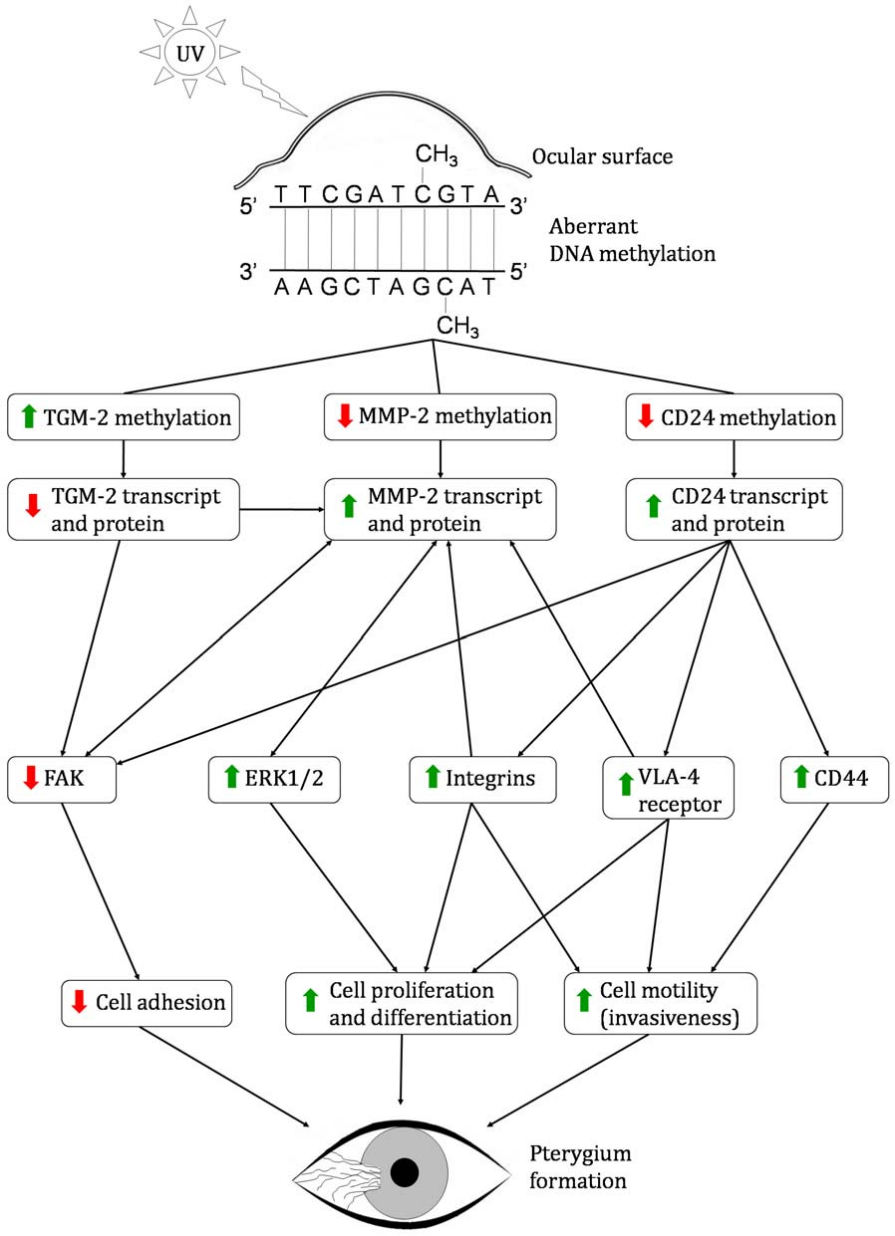
Pterygium pathogenesis. Environmental factors, such as UV exposure, have been known to trigger changes in DNA methylation. UV has also been strongly associated with pterygium formation. This may suggest a mechanism whereby UV can trigger tissue and temporal specific changes in matrix remodeling and cell adhesion related genes, such as transglutaminase 2 (TGM-2), matrix metalloproteinase 2 (MMP-2), and CD24 to initiate pterygium formation. ERK1/2, FAK and VLA-4 are the abbreviations of extracellular signal-regulated kinase 1/2, focal adhesion kinase and very late antigen-4, respectively.

Pterygium formation has been reportedly linked to UV radiation [1], [48]. UV has also been known to trigger changes in the action of methylating and demethylating enzymes [49], [50]. We therefore propose a mechanism whereby environmental influences, such as UV radiation can trigger tissue and temporal specific changes in genes to initiate pterygium (illustrated in **Figure 5**).

Clinically, pterygium is primarily treated by surgical excision. However, aggressive recurrences after surgery are fairly common [51]. Profiling of methylation status of key matrix genes will not only provide clues to pathogenesis but may also have prognostication value. Further studies are now required to establish this. In addition, further work on the methylation status of the TGM-2, MMP-2 and CD24 genes in other multi-factorial human non-neoplastic diseases such as diseases involving scarring or aberrant wound healing are warranted.

In conclusion, we have found aberrant DNA methylation to be present in several key genes associated with wound healing processes in pterygium. In our study, we found that critical CpG islands associated with the MMP-2 and CD24 genes were demethylated in pterygium, whereas TGM-2 gene sequences were over-methylated. These results suggest that the pathogenesis of pterygium may be related to the methylated state of pivotal wound healing and developmental genes.

## Materials and Methods

### Ethics statement

The following protocols conformed to the tenets of the Declaration of Helsinki and were approved by the institutional review board of the Singapore Eye Research Institute, and the written informed consent was acquired from all participating patients.

### Specimens from patients

The surgical excision and procurement of pterygium and conjunctiva tissues from patients was carried out as previously described [52]. Briefly, the pterygium tissue from a patient was compared with the uninvolved conjunctival tissue obtained from the same eye that was excised from the superior temporal quadrant of the bulbar conjunctiva, adjacent to the position of the harvested conjunctival graft.

### Cell culture and treatment

IOBA-NHC were a gift from Yolanda Diebold at the University of Valladolid, Spain. These cells were cultured in a medium containing equal volumes of Dulbecco modified Eagle medium (DMEM) and F12, with an additional 2 ng/ml recombinant human EGF (R&D Systems, Minneapolis, MN), 1 µg/ml bovine insulin, 0.1 µg/ml cholera toxin, 0.5 µg/ml hydrocortisone, and 10% FBS (all except EGF are from Invitrogen-GIBCO, Carlsbad, CA). After reaching 80–90% confluency, the cells were treated with 1, 2 or 3 mM of 5-aza-dC (Sigma, St. Louis, MO) for 24 hours. Some of the cells were left untreated.

### Genomic region selection and DNA methylation analysis

The surgical samples were ground in liquid nitrogen with pestle and mortar after fine chopping. Extraction and purification of DNA from tissues was performed using the Genomic DNA Extraction Kit (Norgen Biotek Corporation, Thorold, Canada). Quality control was ascertained by ensuring that the ratio (260 nm∶280 nm) of the absorbance of the extracted DNA to be 1.80–1.95. Three samples of pterygium tissues and three un-involved conjunctiva tissues from 3 different patients were analysed in this study.

The EpiTYPER Sequenom Mass Array [53] service was provided by Genetic Services, Sequenom, Inc. San Diego, CA. For each sample, 1.25 µg of DNA in a volume of 25 µL was sent to the service. Briefly, this is a previously validated, highly accurate quantitative assay [54] based on base-specific cleavage and mass spectrometry [55] after bisulfite treatment which converted all non-methylated cytosine bases to uracil but with all methylated cytosine bases remain cytosine [56]. This method of assaying for DNA methylation status has been employed in lung cancer [57], profiling of various cancer cell lines [58], developmental changes [31], and differences in tissue function and differentiation [59]. Primers were designed for 48 sequences for 29 matrix and cell adhesion related genes out of 36 genes, which were initially selected for this study. Primers for the remaining 7 genes did not yield satisfactory results and were excluded from the analysis.

Previously, we have performed a microarray analysis to examine the transcript levels of >23000 genes using the Affymetrix U133A Genechip [24], and the data have been made available to the general public (GEO, http://www.ncbi.nlm.nih.gov/gds/ with GEO series accession number GSE2513). The methodology of this study has been reported elsewhere [24]. Genes with associated differential methylation of CpGs were compared against the transcript changes detected on the gene expression data from the Genechip. Upregulated transcripts were considered to be consistent with hypomethylation and vice versa. Subsequent analysis and results presented were focused on the methylation changes that were consistent with differential gene expression.

The sequences for some of the more significant sequences are shown in Table 2. The median amplicon length was 389 bp (min 143, max 650). The median number of CpG units per amplicon was 12 (min 3, max 44). Preliminary quality control (QC) steps were applied to the data and the CpG units that yielded data in greater than 25% of the samples passed initial QC. Data samples that yielded data in greater than 80% for all CpG units within an amplicon were passed for that sample/amplicon pair. CpG units which had data available for less than 25% of all samples were excluded.

**Table 2:** Genomic regions analyzed for DNA methylation.

Gene promoter	NCBI Accession	Description	Start*	End*
CD24_01**	NT_011875.12	Homo sapiens chromosome Y genomic contig,	7355993	7356344
		GRCh37.p2 reference primary assembly.		
		Length = 10102850		
		Features flanking this part of subject sequence:		
		Signal transducer CD24 precursor		
CD24_02**	As above	As above	7356345	7355958
MMP-2	NT_010498.15	Homo sapiens chromosome 16 genomic contig,	9127643	9128045
		GRCh37.p2 reference primary assembly.		
		Length = 42003582		
		Features flanking this part of subject sequence:		
		72 kDa type IV collagenase isoform a preprotein		
TGM-2	NT_011362.9	Bottom of Form Homo sapiens chromosome 20 genomic contig,	6989521	6989864
		GRCh37.p2 reference primary assembly.		
		Length = 31409461		
		Features flanking this part of subject sequence:		
		Protein-glutamine gamma-glutamyltransferase 2 isoform a		
		Protein-glutamine gamma-glutamyltransferase 2 isoform b		

*Refers to position of target sequence in the sequence with the NCBI Accession mentioned.

**More than one EpiTYPER sequences were used for these promoters.

To visualize the proximity of the significant CpG islands from the transcription and translation start sites, alignment of the EpiTYPER target sequence with the appropriate NCBI nucleotide sequence using ClustalW2 software (http://www.ebi.ac.uk/Tools/clustalw2/index.html) was performed.

### Real-time polymerase chain reaction

Primers shown in Table 3 were used for the detection of TGM-2, MMP-2, and CD24 transcript. Reverse transcription of 1 µg of RNA for each sample was performed as previously described [60]. RT-PCR was performed by using the Lightcycler 480 System (Roche Diagnostics, Basel, Switzerland). For this reaction, pre-synthesized hydrolysis FAM (excitation wavelength of 483–533 nm) probe was selected from the Universal ProbeLibrary set and used, based on the ProbeFinder web-based assay (https://www.roche-applied-science.com/sis/rtpcr/upl/index.jsp?id=UP030000) design tool selecting for intron spanning assays. Glyceraldehyde-3-phosphate dehydrogenase (GAPDH) was used as the internal control. For each sample, triplicate wells were used. ΔCt was calculated by subtracting the Ct of GAPDH from the Ct of the targeted gene. The untreated IOBA-NHC cells and uninvolved conjunctiva were considered as the calibrator to compare the relative abundance of TGM-2, MMP-2, and CD24 gene transcript in IOBA-NHC cells treated with 5-aza-dC and in pterygium tissue, respectively. The fold change was determined by the formula 2^(−ΔΔCt)^, where ΔΔCt = ΔCt_sample_−ΔCt_calibrator_.

**Table 3:** Primers used in real-time PCR.

	NCBI	
Gene	Accession No.	Primer Sequence
TGM-2	NM_004613.2	F: AGG GTG ACA AGA GCG AGAT G
		R: TGG TCA TCC ACG ACT CCA C
MMP-2	NM_005430.4	F: ATA ACC TGG ATG CCG TCG T
		R: AGG CAC CCT TGA AGA AGT AGC
CD24	NM_013230.2	F: CCA ACT AAT GCC ACC ACC A
		R: GTG AGA CCA CGA AGA GAC TGG
GAPDH	AF261085.1	F: AGC CAC ATC GCT GAG ACA
		R: GCC CAA TAC GAC CAA ATC C

### Immunofluorescent staining

Conjunctival tissues from normal conjunctival epithelium and pterygium were sectioned with a Microm HM550 cryostat (Microm, Walldorf, Germany) at 8 µm thickness. Sections were fixed with 4% paraformaldehyde for 10 minutes, washed with 1X PBS, and blocked with 4% BSA in 1X PBS containing 0.1% Triton X-100 for one hour. Rabbit polyclonal antibodies against TGM-2 (Abcam, Cambridge, UK) and MMP-2 (Abbiotec, San Diego, CA), and mouse monoclonal antibody against CD24 (Neomarkers, Fremont, CA) were diluted with the blocking buffer at a factor of 1∶150, 1∶200, and 1∶100, respectively and incubated at 4°C overnight. After washing with 1X PBS, the sections were incubated with Alexa Fluor 488-conjugated secondary antibody (Invitrogen) at room temperature for 1 hour. Slides were then mounted with UltraCruz Mounting Medium containing DAPI (Santa Cruz Biotechnology, Santa Cruz, CA). For negative controls, non-immune serum was used in place of the specific primary antibody. Sections were observed under and imaged with a Zeiss Axioplan 2 fluorescence microscope (Zeiss, Oberkochen, Germany).

### In situ zymography

In situ zymography was performed to localize the gelatinase activity in pterygium and conjunctiva cryosections using a previous reported method [61]. Briefly, sections were incubated at room temperature for 2 hours with reaction buffer (0.05 M TrisHCl, 0.15 M NaCl, 5 mM CaCl2, and 0.2 mM NaN3, pH 7.6) containing 40 mg/ml FITC-labeled DQ gelatin, which was available in a gelatinase/collagenase assay kit (EnzChek, Invitrogen). As a negative control, 50 mM 1,10-phenanthroline, a MMP inhibitor, was added to the reaction buffer before applying the FITC-conjugated DQ gelatin to frozen sections. Proteolysis of the FITC-labeled DQ gelatin substrate yields cleaved gelatin-FITC peptides that are fluorescent. The localization of fluorescence indicates the sites of net gelatinolytic activity. After incubation, the sections were washed three times with 1X PBS for 5 min, counterstained with UltraCruz Mounting Medium containing DAPI (Santa Cruz Biotechnology), and coverslip was applied. Localization of gelatinolytic activity of MMPs was viewed and imaged with a Zeiss Axioplan 2 fluorescence microscope (Zeiss).

### Western blot

Normal conjunctiva and pterygium tissue were homogenized individually into RIPA lysis buffer (Santa Cruz Biotechnology). Insoluble materials were removed by 15-minute centrifugation, 10,000 g at 4°C. An equal volume of 4x SDS loading buffer was added to each sample which was then subjected to boiling for 5 min at 99°C. The sample was then left on ice for 10 minutes before reduction on a sodium dodecyl sulfate-polyacrylamide gel electrophoresis (SDS-PAGE-gradient 10%). The amounts of protein applied was 40 µg. Resolved proteins were electrophoretically transferred onto a nitrocellulose membrane and blocked in 1X TBS containing 5% non-fat milk, followed by overnight incubation with the rabbit polyclonal antibodies against TGM-2 (Abcam; 1∶500) and MMP-2 (Abbiotec; 1∶1000), and mouse monoclonal antibody against CD24 (Neomarkers; 1∶500) in 4°C with agitation. The membranes were then washed vigorously three times each for five minutes in 1X TBS, 0.1% Tween-20. The HRP-conjugated secondary antibodies (Santa Cruz Biotechnology) were then applied at a dilution of 1∶2000. Immunoreactivity was visualized with Super Signal West Pico chemiluminescence reagent (Pierce Biotechnology, Rockford, IL).

### Statistical analysis

Student's *t*-test or analysis of variance (ANOVA) followed by the least significant difference (LSD) test were used to determine the difference between groups. *p*<0.05 was accepted as significantly different.

## Supporting Information

Table S1 Entities that were analyzed in the pathways shown in Figure 4
(0.04 MB DOC)

Table S2 Relationship between the entities analyzed in the pathways shown in Figure 4
(0.05 MB DOC)

Figure S1 Hypermethylation of transglutaminase 2 (TGM-2) promoter in the pterygium. The brackets indicate hypermethylated CpG sites in pterygium (shown in Table 1). CGs highlighted in grey represent CpG units that were not significantly methylated or differentially methylated but not contributing to the dysregulation of TGM-2 transcripts.
(0.19 MB TIF)

Figure S2 Hypomethylation of matrix metalloproteinase 2 (MMP-2) promoter in the pterygium. The bracket indicates the hypomethylated CpG site in pterygium (shown in Table 1). CGs highlighted in grey represents the other CpG units that were tested and found to be not significantly methylated.
(0.34 MB TIF)

Figure S3 Hypomethylation of CD24 promoter in the pterygium. More than one EpiTYPER sequences were used for this promoter: (A) CD24_01. (B) CD24_02. The brackets indicate the differentially methylated CpG sites that were shown in Table 1. CGs highlighted in grey show the other CpG units that were not differentially methylated.
(0.93 MB TIF)
