# Correction: Aberrant DNA Methylation of Matrix Remodeling and Cell Adhesion Related Genes in Pterygium

**DOI:** 10.1371/annotation/814c14d4-eee6-44e2-bea8-cac11a0bae8f

**Published:** 2011-03-04

**Authors:** Andri K. Riau, Tina T. Wong, Sharon N. Finger, Shyam S. Chaurasia, Ai Hua Hou, Silin Chen, Shang Juan Yu, Louis Tong

Wanwen Lan was not included in the byline. This author should be listed as the third author and affiliated with Ocular Wound Healing and Therapeutics Laboratory, Singapore Eye Research Institute, Singapore, Singapore. The correct citation is: Riau AK, Wong TT, Lan W, Finger SN, Chaurasia SS, Hou AH, et al. (2011) Aberrant DNA Methylation of Matrix Remodeling and Cell Adhesion Related Genes in Pterygium. PLoS ONE 6(2): e14687. doi:10.1371/journal.pone.0014687. The correct author contributions are: Conceived and designed the experiments: AKR TW LT. Performed the experiments: AKR SF SC SJY. Analyzed the data: AKR TW SF SSC AHH SC SJY LT. Contributed reagents/materials/analysis tools: TW WL SSC LT. Wrote the paper: AKR WL LT.

