# Supplementary material for: Aberrant DNA Methylation of Matrix Remodeling and Cell Adhesion Related Genes in Pterygium
Source: PLoS One. 2011 Feb 16;6(2):e14687. doi: 10.1371/journal.pone.0014687 (PMC3040179; doi:10.1371/journal.pone.0014687)
Supplement: Table S1 — Entities that were analyzed in the pathways shown in Figure 4 (0.04 MB DOC) [file pone.0014687.s001.doc]

**Table S1.** Entities that were analyzed in the pathways shown in Figure 4

| **Name** | **Type** | **Description** | **Notes** |
| --- | --- | --- | --- |
| CD24 | Protein | CD24 molecule | This gene encodes a sialoglycoprotein that is expressed on mature granulocytes and in many B cells. The encoded protein is anchored via a glycosyl phosphatidylinositol (GPI) link to the cell surface. An alignment of this gene's sequence finds genomic locations with similarity on chromosomes 3p26, 15q21, 15q22, 20q11.2 and Yq11.1. Whether transcription, and corresponding translation, occurs at each of these other genomic locations needs to be experimentally determined. |
| CD44 | Protein | CD44 antigen | Adhesion molecule involved in migration, cell fusion and resportion in osteoclasts that also plays a role in cellular metastasis |
| CSF2 | Protein | Colony stimulating factor 2 (granulocyte-macrophage) | Plays a role in alveolar epithelial fluid transport |
| E-cadherin | Protein | Cadherin 1 | Cell-cell adhesion molecule; may play a role in axonal growth and synase formation |
| ERK1/2 | Functional Class | Extracellular signal-regulated kinase 1/2 |  |
| FAK | Protein | Focal adhesion kinase (Protein tyrosine kinase 2) | Tyrosine kinase that is enriched in focal adhesions; may regulate mechanical signal transduction in cardiac myocytes |
| IGFBP2 | Protein | Insulin-like growth factor binding protein 2 | Protein that binds to Igf-I and Igf-II |
| ITG | Functional Class | Integrins |  |
| MMP2 | Protein | Matrix metalloproteinase 2 | Plays important roles in wound healing, angiogenesis, platelet aggregation, and tumor metastasis; mediates the response of the intrinsic glomerular mesangial cell to inflammatory stimuli |
| p38 MAPK | Protein | Mitogen activated protein kinase 14 | Mitogen-activated protein kinase; involved in intracellular signalling, inhibition of apoptosis and gene activation |
| PDGF | Complex | Platelet-derived growth factor |  |
| PKC | Protein | Mitogen activated protein kinase 3 | Kinase involved in intracellular signalling; component of Mapk signalling pathway |
| Protein tyrosine kinase | Functional Class | |  |
| SRC | Protein | Rous sarcoma oncogene | Mediates growth effects of both the precursor and fully processed forms of gastrins on rat intestinal epithelial cells |
| TGM2 | Protein | Transglutaminase 2, C polypeptide | Catalyzes acyl transferase reaction of gamma-carboxamide groups of glutamine residues (donor peptide) with epsilon-amino groups of lysine residues (acceptor peptide) |
| VCAM1 | Protein | Vascular cell adhesion molecule 1 | Binds integrin very late antigen (VLA4); plays a role in cell adhesion |
| VLA-4 receptor | Complex | Very late antigen-4 receptor |  |
