# Supplementary material for: Aberrant DNA Methylation of Matrix Remodeling and Cell Adhesion Related Genes in Pterygium
Source: PLoS One. 2011 Feb 16;6(2):e14687. doi: 10.1371/journal.pone.0014687 (PMC3040179; doi:10.1371/journal.pone.0014687)
Supplement: Table S2 — Relationship between the entities analyzed in the pathways shown in Figure 4 (0.05 MB DOC) [file pone.0014687.s002.doc]

**Table S2.** Relationship between the entities analyzed in the pathways shown in Figure 4

| **Relation** | **Type** | **MedLine Reference** |
| --- | --- | --- |
| CD24 --+> CD44 | Expression | 11359815 |
| CD24 --+> E-cadherin | Expression | 16930538 |
| CD24 --+> ITG | Regulation | 7534304, 16322224 |
| CD24 --+> VLA-4 receptor | Expression | 8145052 |
| CD24 ---- Protein tyrosine kinase | Binding | 8753773 |
| CD24 ---- SRC | Binding | 16322224 |
| CD24 ---> VCAM1 | Regulation | 7534304 |
| CD24 ---| FAK | Regulation | 16322224 |
| CD44 ---| MMP2 | Expression | 12393872, 11564733 |
| CSF2 --+> CD24 | Expression | 12193691 |
| CSF2 --+> MMP2 | Expression | 10840163, 16912178, 10233890 |
| E-cadherin ---| MMP2 | Regulation | 10446959 |
| ERK1/2 --+> MMP2 | Expression | 17652746, 16498067, 15509661, 16284240, 15611113 |
| FAK ---- TGM2 | Binding | 17079475 |
| FAK ---| MMP2 | Expression | 16424009 |
| IGFBP2 --+> MMP2 | Expression | 12907597, 15643522 |
| IGFBP2 ---| CD24 | Expression | 17475624 |
| ITG --+> MMP2 | Expression | 17283249, 12441353, 11331272, 11564733, 11872628 |
| ITG ---- MMP2 | Binding | 11289157, 11912174, 15665286, 16230401 |
| ITG ---- TGM2 | Binding | 17314516, 16818508 |
| MMP2 --+> ERK1/2 | Regulation | 16951163 |
| MMP2 ---> ITG | Expression | 10667602 |
| MMP2 ---> p38 MAPK | Regulation | 15492828 |
| MMP2 ---| CD44 | Expression | 12393872, 16035618 |
| MMP2 ---| FAK | Regulation | 16148033, 12620921 |
| MMP2 ---| SRC | Regulation | 12963732 |
| p38 MAPK --+> MMP2 | Expression | 16450376, 15492828 |
| PDGF --+> MMP2 | Molecular synthesis | 10704614 |
| PDGF ---> CD24 | Regulation | 15994924 |
| PKC --+> MMP2 | Expression | 11174068, 15851572 |
| Protein tyrosine kinase --+> MMP2 | Expression | 15219830 |
| SRC --+> MMP2 | Regulation | 14617636, 12947321, 16453304, 15862818 |
| SRC --+> MMP2 | Expression | 16453304 |
| SRC ---> MMP2 | Molecular transport | 16951163 |
| TGM2 --+> FAK | Regulation | 17079475 |
| TGM2 --+> MMP2 | Expression | 15199098 |
| TGM2 ---> ERK1/2 | Regulation | 18499669, 15556610 |
| TGM2 ---> PKC | Regulation | 17507797 |
| VCAM1 ---> MMP2 | Regulation | 10505981, 15265790 |
| VLA-4 receptor --+> MMP2 | Expression | 10633824 |

--+> = upregulation

---| = downregulation

---> = regulation which does not directly result in up- or downregulation of the target molecule

---- = binding
