# Supplementary figures and images for: Aberrant DNA Methylation of Matrix Remodeling and Cell Adhesion Related Genes in Pterygium
Source: PLoS One. 2011 Feb 16;6(2):e14687. doi: 10.1371/journal.pone.0014687 (PMC3040179; doi:10.1371/journal.pone.0014687)

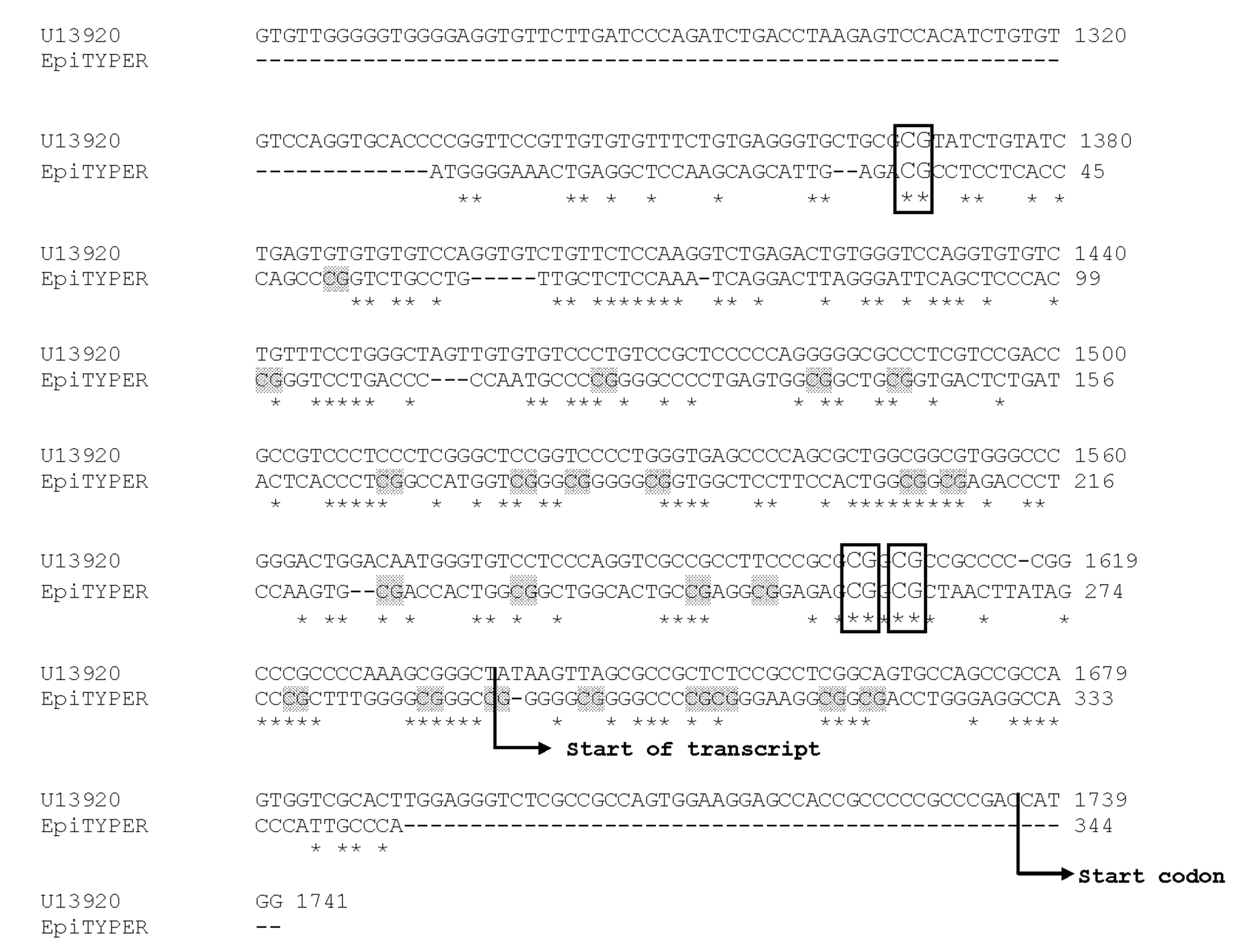

Supplement: Figure S1 — Hypermethylation of transglutaminase 2 (TGM-2) promoter in the pterygium. The brackets indicate hypermethylated CpG sites in pterygium (shown in Table 1). CGs highlighted in grey represent CpG units that were not significantly methylated or differentially methylated but not contributing to the dysregulation of TGM-2 transcripts. (0.19 MB TIF) [file pone.0014687.s003.tif]

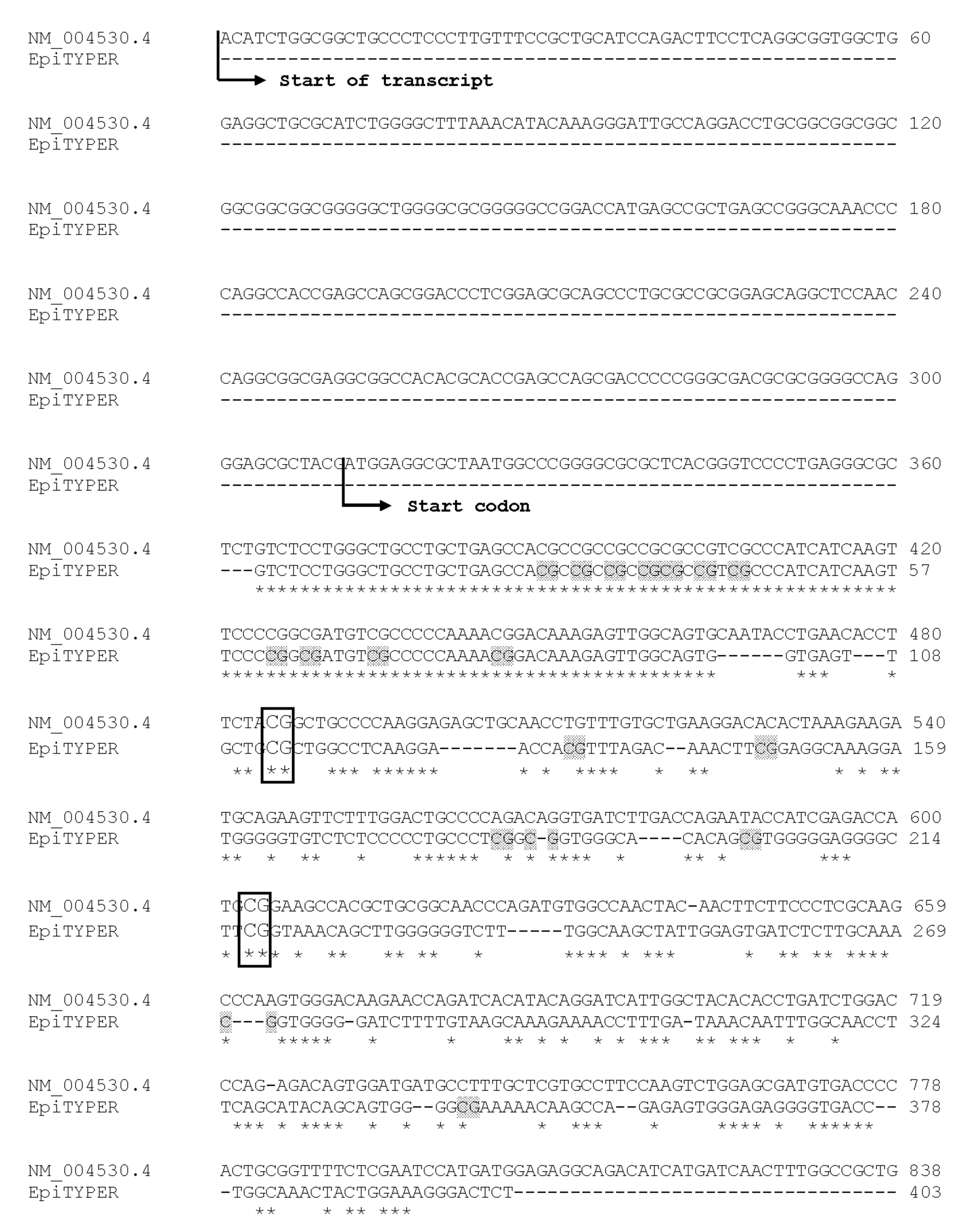

Supplement: Figure S2 — Hypomethylation of matrix metalloproteinase 2 (MMP-2) promoter in the pterygium. The bracket indicates the hypomethylated CpG site in pterygium (shown in Table 1). CGs highlighted in grey represents the other CpG units that were tested and found to be not significantly methylated. (0.34 MB TIF) [file pone.0014687.s004.tif]

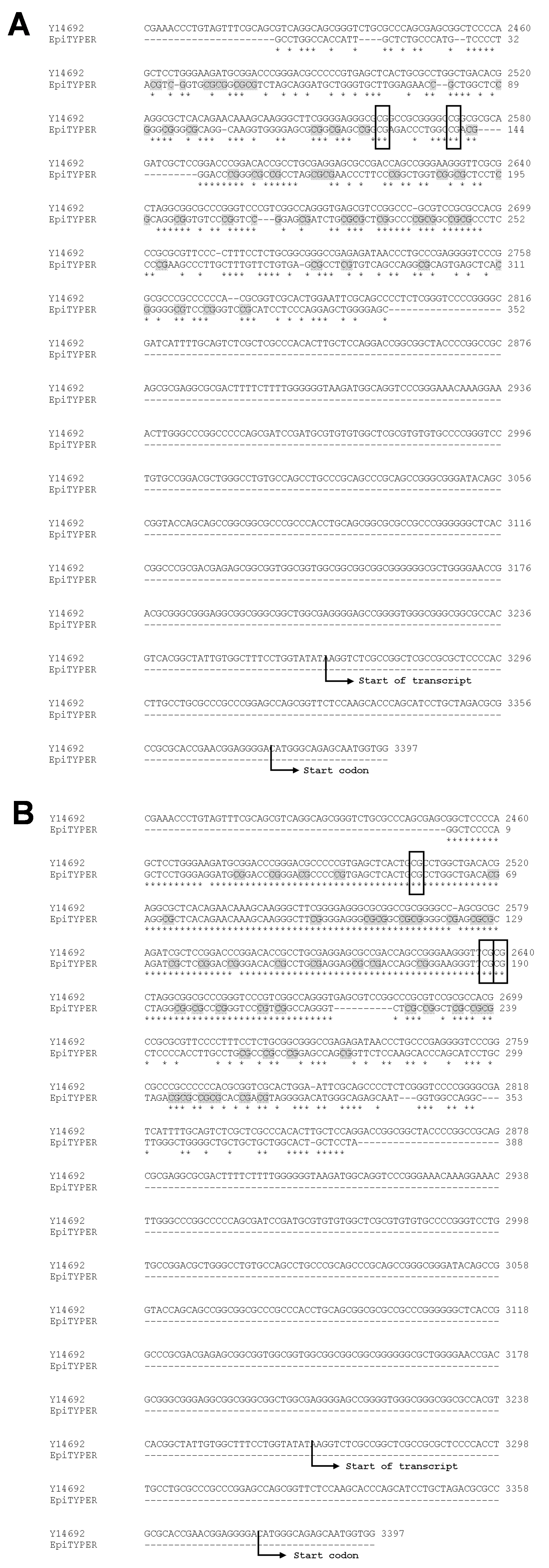

Supplement: Figure S3 — Hypomethylation of CD24 promoter in the pterygium. More than one EpiTYPER sequences were used for this promoter: (A) CD24_01. (B) CD24_02. The brackets indicate the differentially methylated CpG sites that were shown in Table 1. CGs highlighted in grey show the other CpG units that were not differentially methylated. (0.93 MB TIF) [file pone.0014687.s005.tif]
